# Supplementary material for: Coccomyxa subellipsoidea KJ Components Enhance the Expression of Metallothioneins and Th17 Cytokines during Human T Cell Activation
Source: Microorganisms. 2024 Apr 5;12(4):741. doi: 10.3390/microorganisms12040741 (PMC11051862; doi:10.3390/microorganisms12040741)
Supplement: Supplementary file 1 [file microorganisms-12-00741-s001.zip › Supplemental_Figures.pptx]

## Slide 1
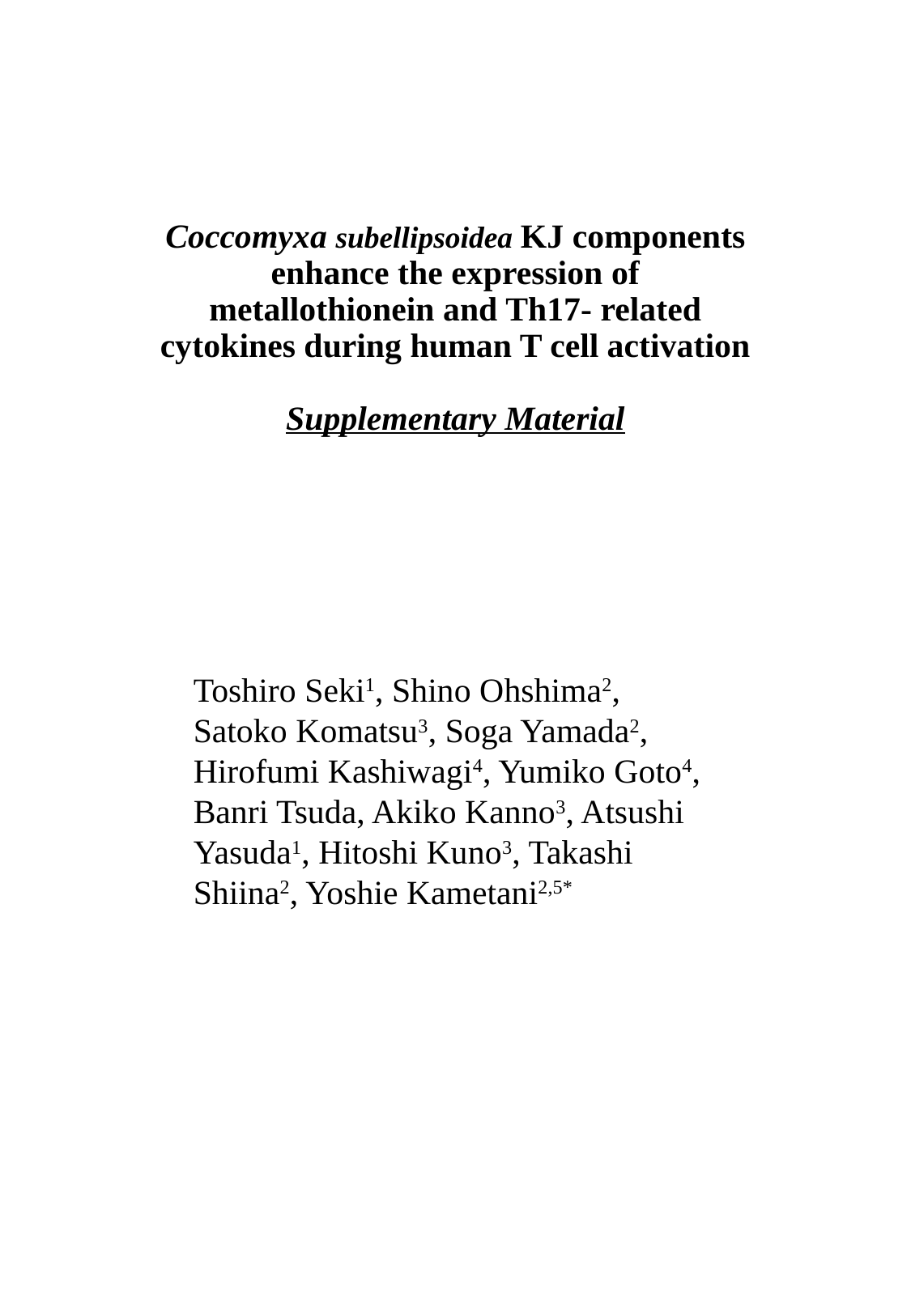

# Coccomyxa subellipsoidea KJ components enhance the expression of metallothionein and Th17- related cytokines during human T cell activation Supplementary Material
Toshiro Seki1, Shino Ohshima2, Satoko Komatsu3, Soga Yamada2, Hirofumi Kashiwagi4, Yumiko Goto4, Banri Tsuda, Akiko Kanno3, Atsushi Yasuda1, Hitoshi Kuno3, Takashi Shiina2, Yoshie Kametani2,5*

## Slide 2
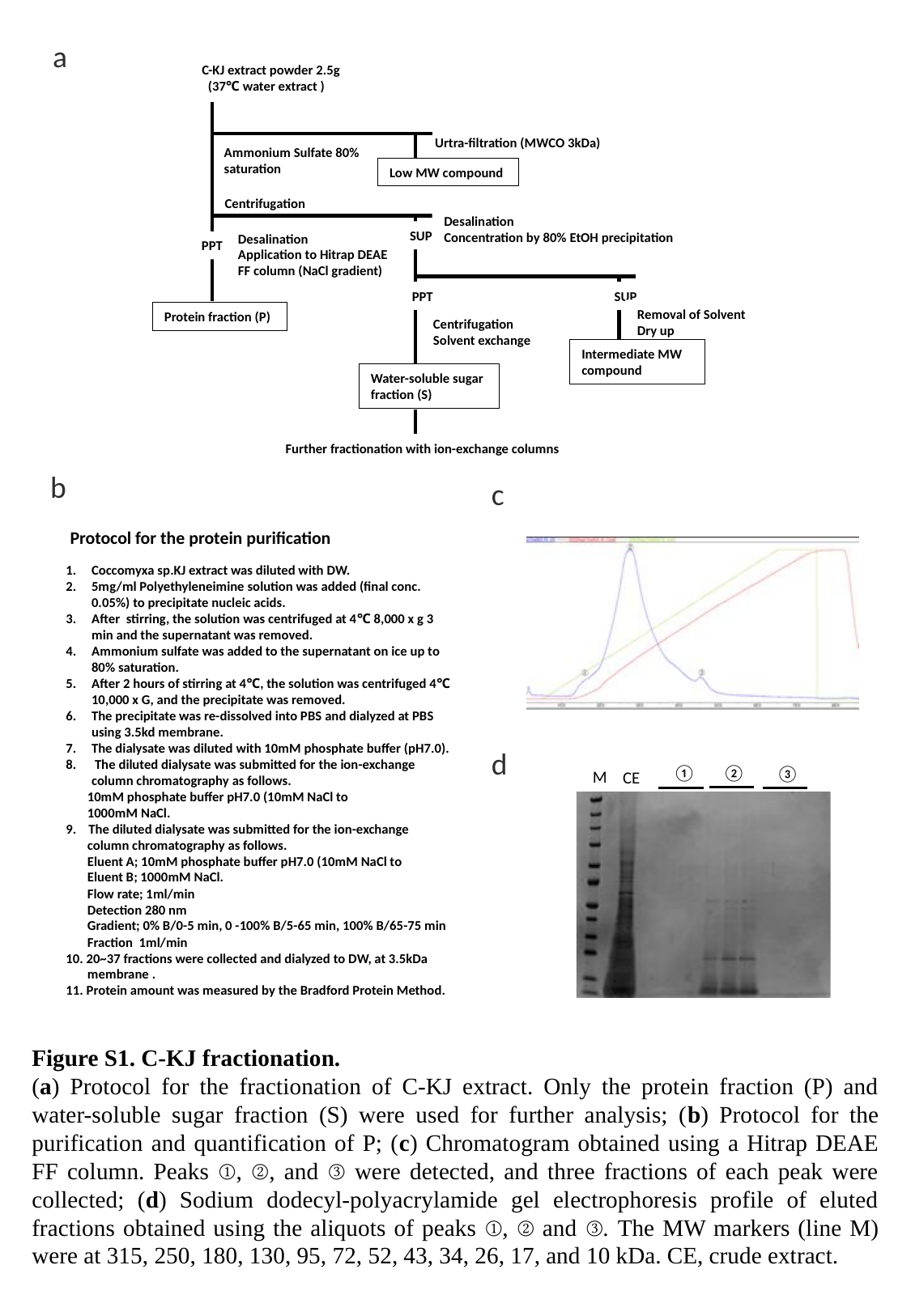

a
C-KJ extract powder 2.5g
 (37℃ water extract )
Urtra-filtration (MWCO 3kDa)
Ammonium Sulfate 80% saturation
Low MW compound
Centrifugation
Desalination
Concentration by 80% EtOH precipitation
SUP
Desalination
Application to Hitrap DEAE FF column (NaCl gradient)
PPT
PPT
SUP
Removal of Solvent Dry up
Protein fraction (P)
Centrifugation
Solvent exchange
Intermediate MW compound
Water-soluble sugar fraction (S)
Further fractionation with ion-exchange columns
 b
 c
 Protocol for the protein purification
Coccomyxa sp.KJ extract was diluted with DW.
5mg/ml Polyethyleneimine solution was added (final conc. 0.05%) to precipitate nucleic acids.
After stirring, the solution was centrifuged at 4℃ 8,000 x g 3 min and the supernatant was removed.
Ammonium sulfate was added to the supernatant on ice up to 80% saturation.
After 2 hours of stirring at 4℃, the solution was centrifuged 4℃ 10,000 x G, and the precipitate was removed.
The precipitate was re-dissolved into PBS and dialyzed at PBS using 3.5kd membrane.
The dialysate was diluted with 10mM phosphate buffer (pH7.0).
 The diluted dialysate was submitted for the ion-exchange column chromatography as follows.
 10mM phosphate buffer pH7.0 (10mM NaCl to
 1000mM NaCl.
9. The diluted dialysate was submitted for the ion-exchange
 column chromatography as follows.
 Eluent A; 10mM phosphate buffer pH7.0 (10mM NaCl to
 Eluent B; 1000mM NaCl.
 Flow rate; 1ml/min
 Detection 280 nm
 Gradient; 0% B/0-5 min, 0 -100% B/5-65 min, 100% B/65-75 min
 Fraction 1ml/min
10. 20~37 fractions were collected and dialyzed to DW, at 3.5kDa
 membrane .
11. Protein amount was measured by the Bradford Protein Method.
 d
①
②
③
M
CE
Figure S1. C-KJ fractionation.
(a) Protocol for the fractionation of C-KJ extract. Only the protein fraction (P) and water-soluble sugar fraction (S) were used for further analysis; (b) Protocol for the purification and quantification of P; (c) Chromatogram obtained using a Hitrap DEAE FF column. Peaks ①, ②, and ③ were detected, and three fractions of each peak were collected; (d) Sodium dodecyl-polyacrylamide gel electrophoresis profile of eluted fractions obtained using the aliquots of peaks ①, ② and ③. The MW markers (line M) were at 315, 250, 180, 130, 95, 72, 52, 43, 34, 26, 17, and 10 kDa. CE, crude extract.

## Slide 3
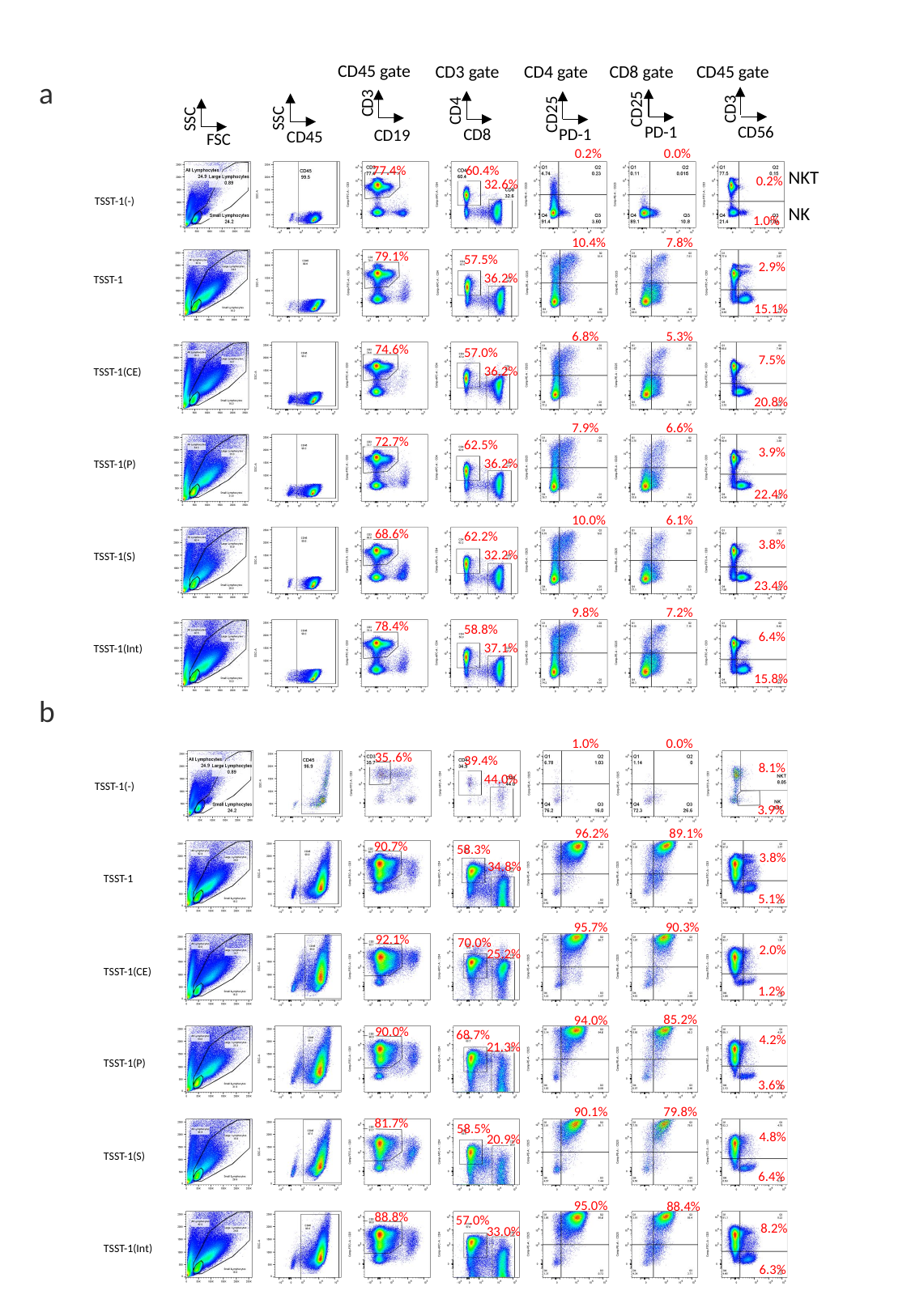

CD45 gate
CD3
CD19
CD3 gate
CD4
CD8
CD8 gate
CD25
PD-1
CD45 gate
CD3
CD56
CD4 gate
CD25
PD-1
 a
SSC
CD45
SSC
FSC
0.0%
0.2%
77.4%
60.4%
NKT
0.2%
32.6%
TSST-1(-)
NK
1.0%
10.4%
7.8%
79.1%
57.5%
2.9%
36.2%
TSST-1
15.1%
6.8%
5.3%
74.6%
57.0%
7.5%
36.2%
TSST-1(CE)
20.8%
7.9%
6.6%
72.7%
62.5%
3.9%
36.2%
TSST-1(P)
22.4%
10.0%
6.1%
68.6%
62.2%
3.8%
32.2%
TSST-1(S)
23.4%
9.8%
7.2%
78.4%
58.8%
6.4%
37.1%
TSST-1(Int)
15.8%
 b
1.0%
0.0%
35..6%
39.4%
8.1%
44.0%
TSST-1(-)
3.9%
96.2%
89.1%
90.7%
58.3%
3.8%
34.8%
TSST-1
5.1%
95.7%
90.3%
92.1%
70.0%
2.0%
25.2%
TSST-1(CE)
1.2%
85.2%
94.0%
90.0%
68.7%
4.2%
21.3%
TSST-1(P)
3.6%
79.8%
90.1%
81.7%
58.5%
4.8%
20.9%
TSST-1(S)
6.4%
95.0%
88.4%
88.8%
57.0%
8.2%
33.0%
TSST-1(Int)
6.3%

## Slide 4
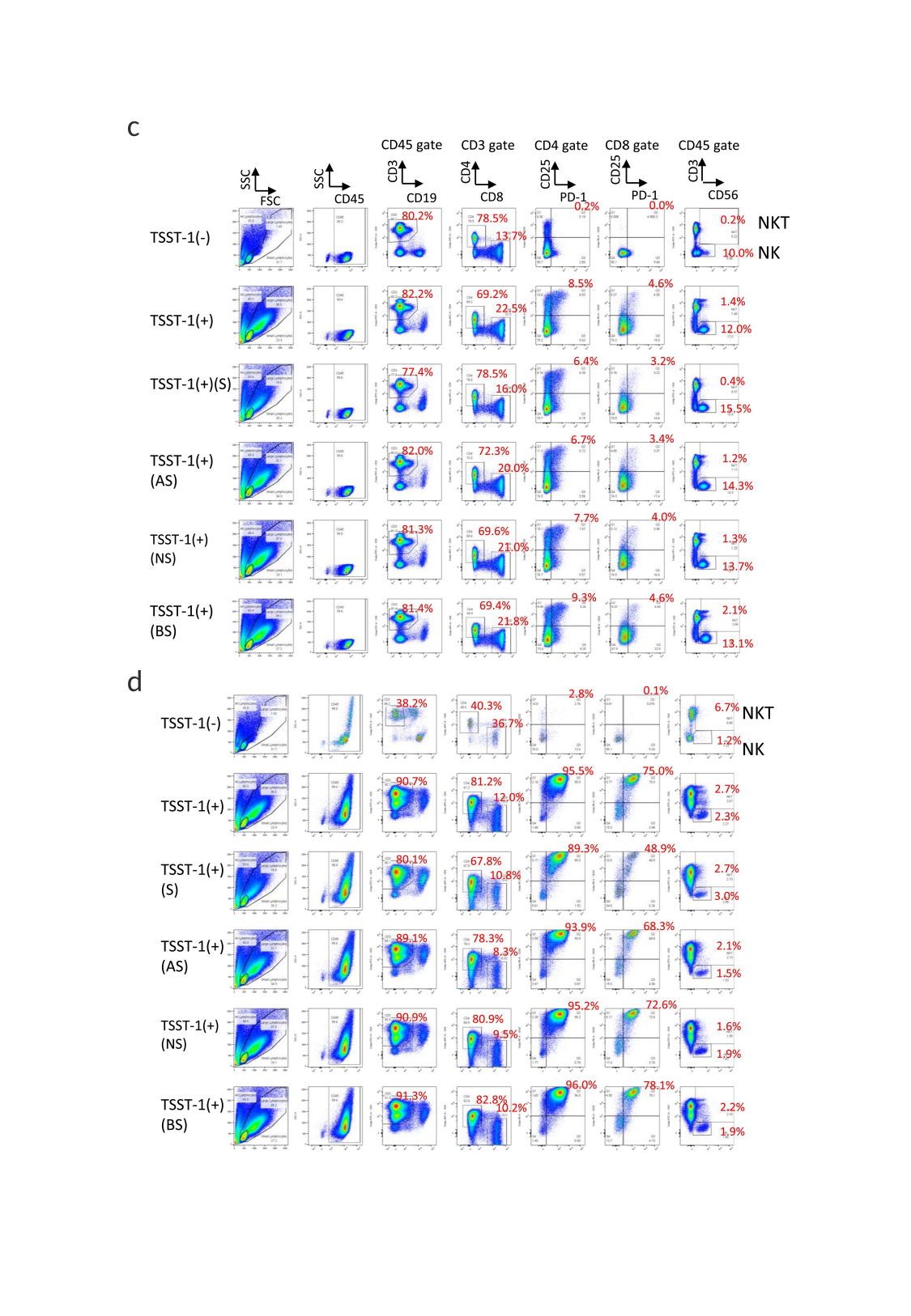

c
 d

## Slide 5
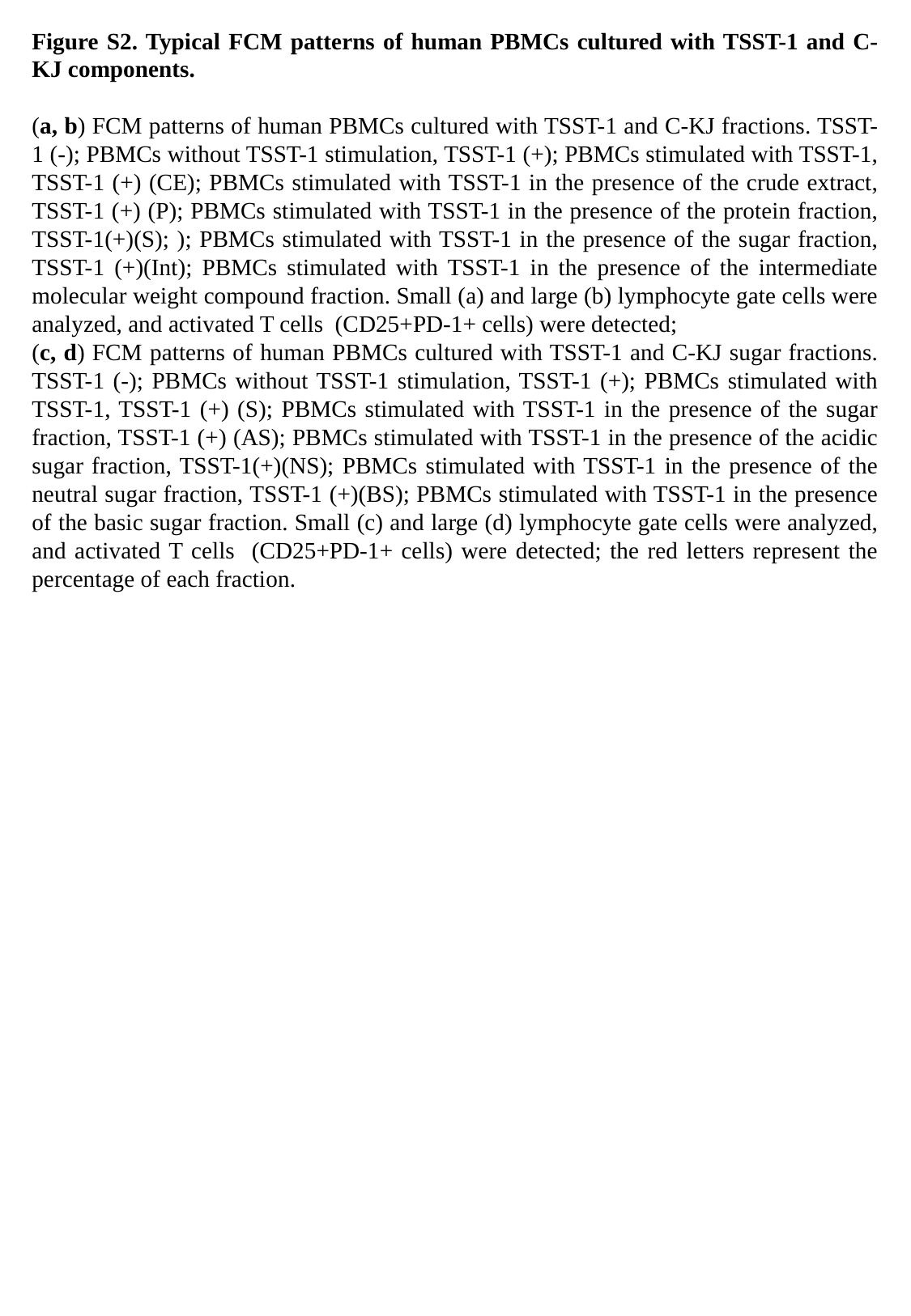

Figure S2. Typical FCM patterns of human PBMCs cultured with TSST-1 and C-KJ components.
(a, b) FCM patterns of human PBMCs cultured with TSST-1 and C-KJ fractions. TSST-1 (-); PBMCs without TSST-1 stimulation, TSST-1 (+); PBMCs stimulated with TSST-1, TSST-1 (+) (CE); PBMCs stimulated with TSST-1 in the presence of the crude extract, TSST-1 (+) (P); PBMCs stimulated with TSST-1 in the presence of the protein fraction, TSST-1(+)(S); ); PBMCs stimulated with TSST-1 in the presence of the sugar fraction, TSST-1 (+)(Int); PBMCs stimulated with TSST-1 in the presence of the intermediate molecular weight compound fraction. Small (a) and large (b) lymphocyte gate cells were analyzed, and activated T cells (CD25+PD-1+ cells) were detected;
(c, d) FCM patterns of human PBMCs cultured with TSST-1 and C-KJ sugar fractions. TSST-1 (-); PBMCs without TSST-1 stimulation, TSST-1 (+); PBMCs stimulated with TSST-1, TSST-1 (+) (S); PBMCs stimulated with TSST-1 in the presence of the sugar fraction, TSST-1 (+) (AS); PBMCs stimulated with TSST-1 in the presence of the acidic sugar fraction, TSST-1(+)(NS); PBMCs stimulated with TSST-1 in the presence of the neutral sugar fraction, TSST-1 (+)(BS); PBMCs stimulated with TSST-1 in the presence of the basic sugar fraction. Small (c) and large (d) lymphocyte gate cells were analyzed, and activated T cells (CD25+PD-1+ cells) were detected; the red letters represent the percentage of each fraction.

## Slide 6
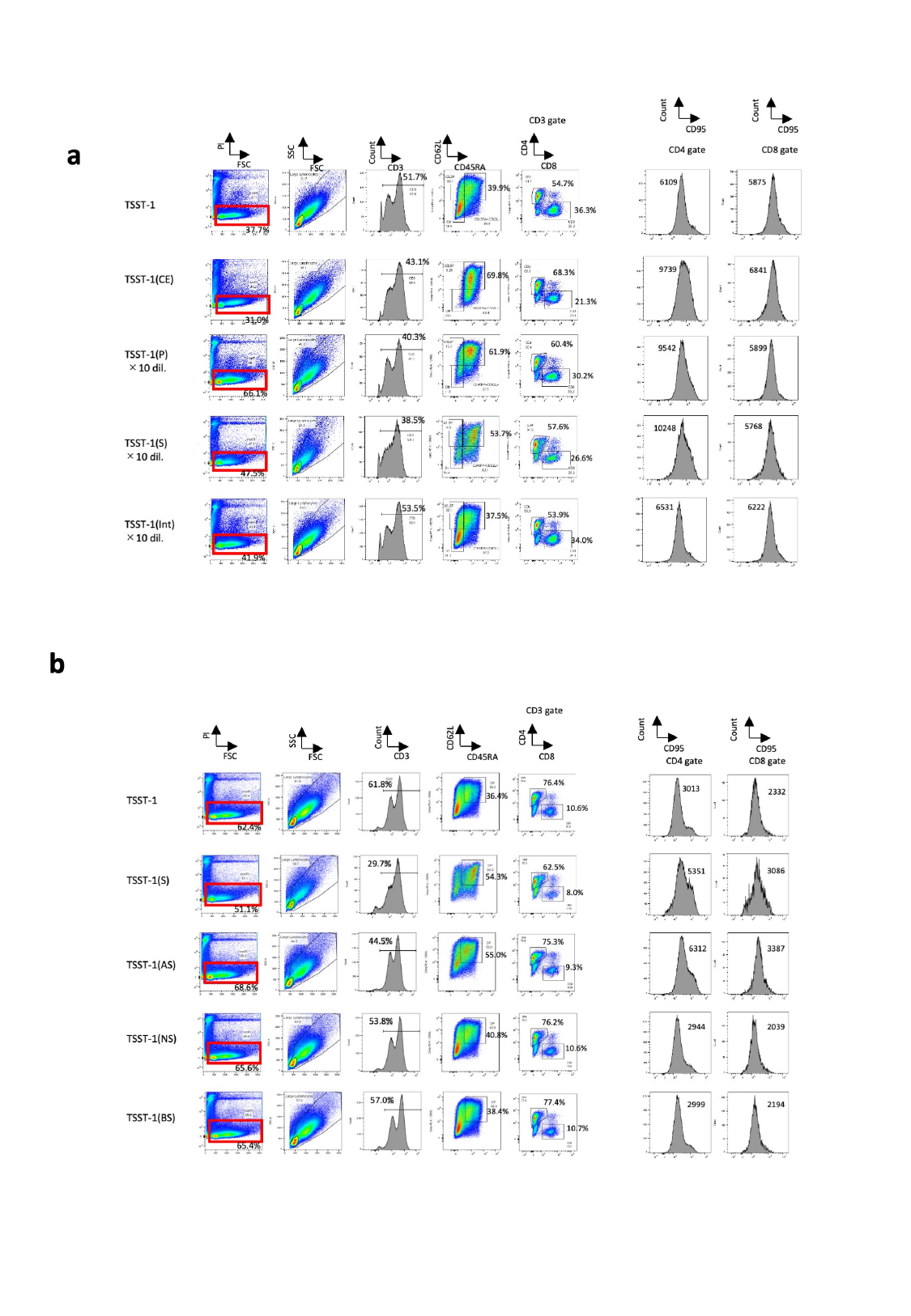

## Slide 7
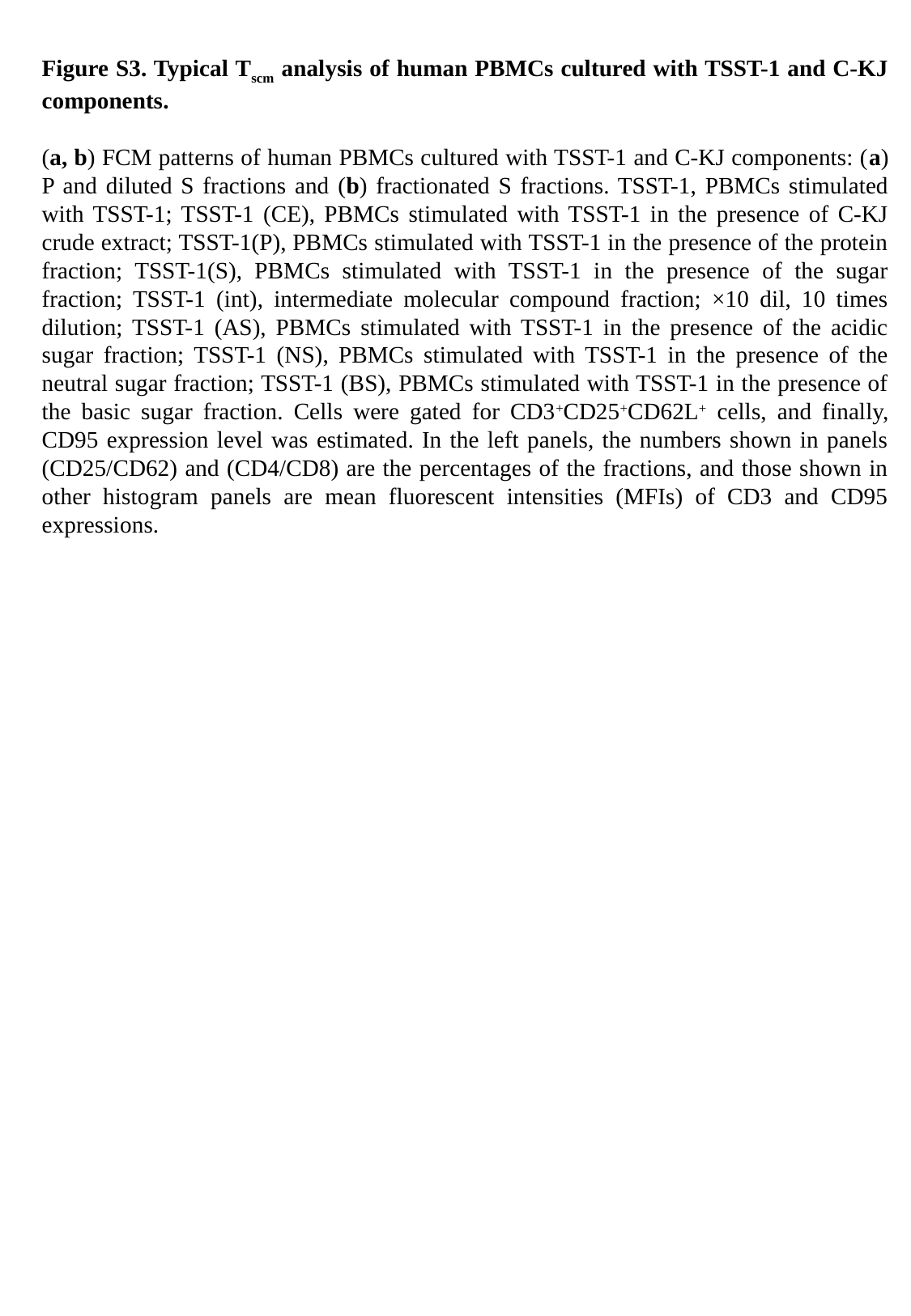

Figure S3. Typical Tscm analysis of human PBMCs cultured with TSST-1 and C-KJ components.
(a, b) FCM patterns of human PBMCs cultured with TSST-1 and C-KJ components: (a) P and diluted S fractions and (b) fractionated S fractions. TSST-1, PBMCs stimulated with TSST-1; TSST-1 (CE), PBMCs stimulated with TSST-1 in the presence of C-KJ crude extract; TSST-1(P), PBMCs stimulated with TSST-1 in the presence of the protein fraction; TSST-1(S), PBMCs stimulated with TSST-1 in the presence of the sugar fraction; TSST-1 (int), intermediate molecular compound fraction; ×10 dil, 10 times dilution; TSST-1 (AS), PBMCs stimulated with TSST-1 in the presence of the acidic sugar fraction; TSST-1 (NS), PBMCs stimulated with TSST-1 in the presence of the neutral sugar fraction; TSST-1 (BS), PBMCs stimulated with TSST-1 in the presence of the basic sugar fraction. Cells were gated for CD3+CD25+CD62L+ cells, and finally, CD95 expression level was estimated. In the left panels, the numbers shown in panels (CD25/CD62) and (CD4/CD8) are the percentages of the fractions, and those shown in other histogram panels are mean fluorescent intensities (MFIs) of CD3 and CD95 expressions.

## Slide 8
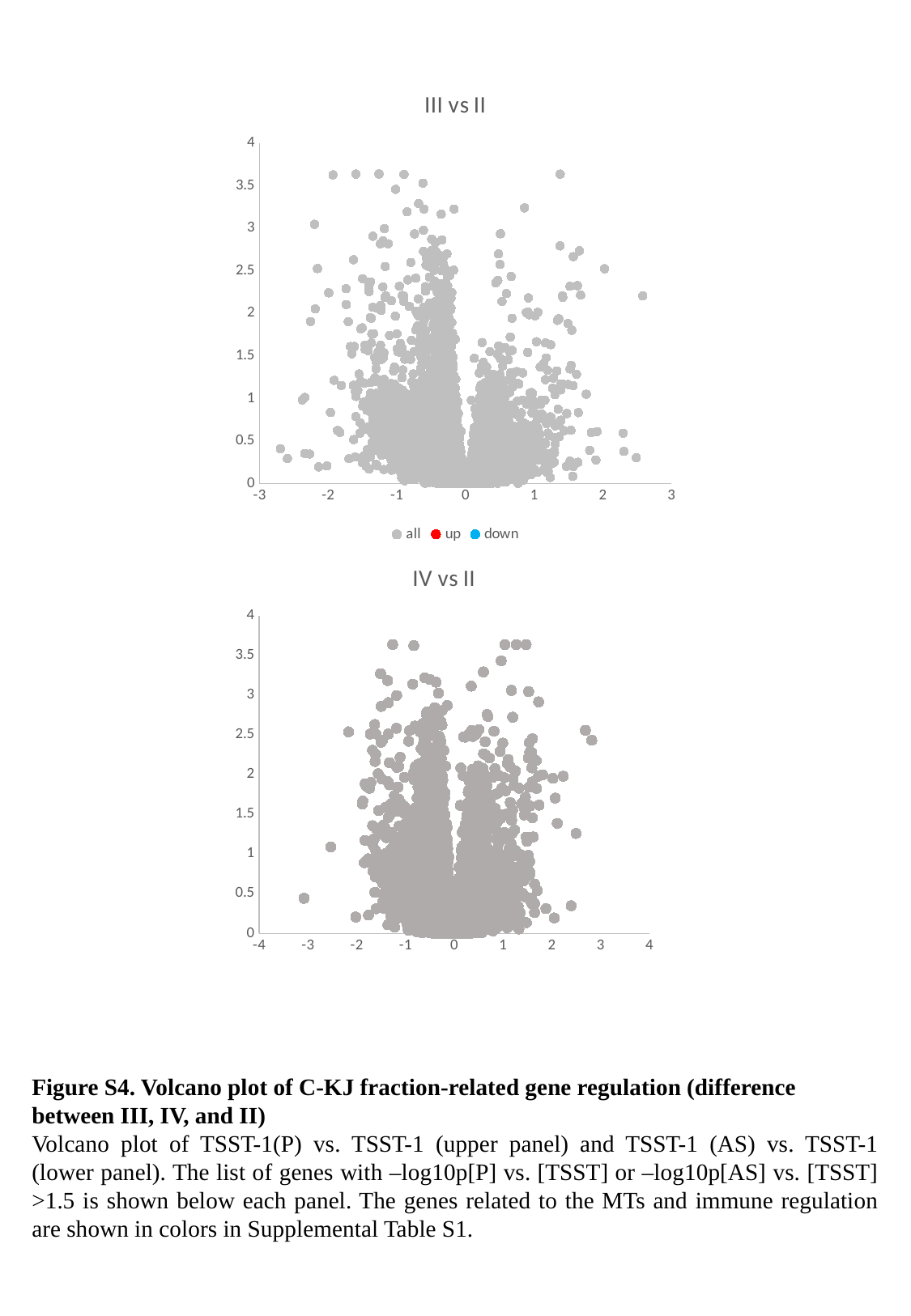

### Chart: III vs II
| Category | all | | |
|---|---|---|---|
### Chart: IV vs II
| Category | -LOG10 ([IV] vs [II]) | -LOG10 ([IV] vs [II]) | -LOG10 ([IV] vs [II]) |
|---|---|---|---|Figure S4. Volcano plot of C-KJ fraction-related gene regulation (difference between III, IV, and II)
Volcano plot of TSST-1(P) vs. TSST-1 (upper panel) and TSST-1 (AS) vs. TSST-1 (lower panel). The list of genes with –log10p[P] vs. [TSST] or –log10p[AS] vs. [TSST] >1.5 is shown below each panel. The genes related to the MTs and immune regulation are shown in colors in Supplemental Table S1.

## Slide 9
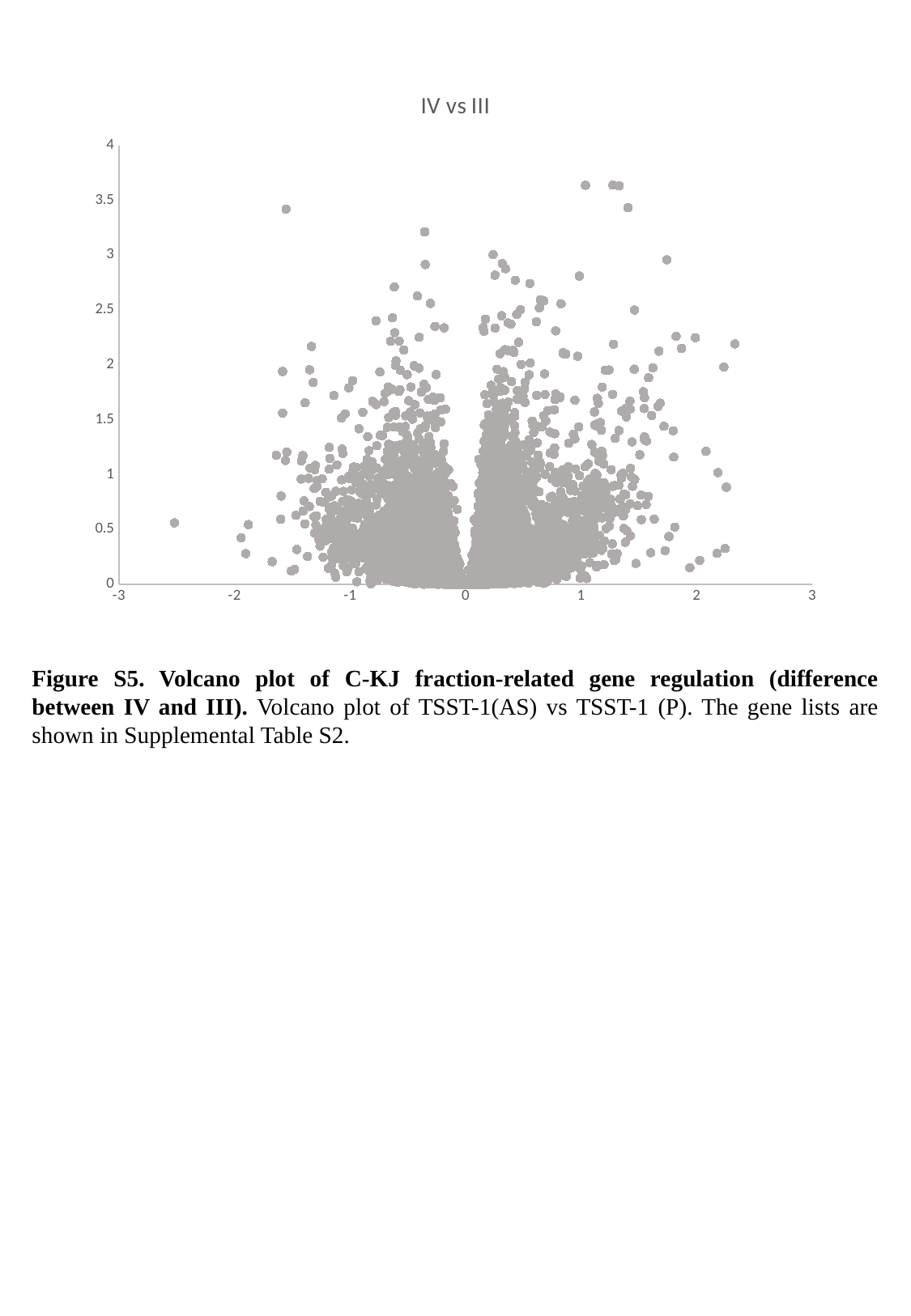

### Chart: IV vs III
| Category | -LOG10 ([IV] vs [III]) | -LOG10 ([IV] vs [III]) | -LOG10 ([IV] vs [III]) |
|---|---|---|---|Figure S5. Volcano plot of C-KJ fraction-related gene regulation (difference between IV and III). Volcano plot of TSST-1(AS) vs TSST-1 (P). The gene lists are shown in Supplemental Table S2.
